# Genomic characterization of non-schistosomiasis-related squamous cell carcinoma of the urinary bladder: A retrospective exploratory study

**DOI:** 10.1371/journal.pone.0259272

**Published:** 2021-12-01

**Authors:** Esmail M. Al-ezzi, Zachary W. Veitch, Samer H. Salah, Theodorus H. Van der Kwast, Tracy L. Stockley, Shamini Selvarajah, Tong Zhang, Srikala S. Sridhar, Adrian G. Sacher, Nazanin Fallah-rad, Girish S. Kulkarni, Alexandre R. Zlotta, Antonio Finelli, Aaron R. Hansen

**Affiliations:** 1 Division of Medical Oncology and Hematology, Princess Margaret Cancer Centre, Toronto, ON, Canada; 2 Division of Medical Oncology and Hematology, King Hussain Cancer Center, Amman, Jordan; 3 Department of Pathology, Toronto General Hospital, Toronto, ON, Canada; 4 Division of Clinical Laboratory Genetics, Laboratory Medicine Program, University Health Network, Toronto, ON, Canada; 5 Division of Urologic Oncology, Princess Margaret Cancer Centre, Toronto, ON, Canada; Sun Yat-sen University, CHINA

## Abstract

**Background:**

Non-schistosomiasis related-squamous cell carcinoma of urinary bladder (NSR-SCCUB) is a rare tumor subtype distinct from urothelial carcinoma (UC). Studies assessing molecular biomarkers in bladder cancer have generally focused on UC, and genomic data of NSR-SCCUB is limited. We aim to provide additional insight into the molecular underpinnings of this rare entity.

**Methods:**

NSR-SCCUB patients were identified retrospectively at Princess Margaret Cancer Centre between 2002 and 2017. Demographics, disease characteristics, therapeutic approaches, and outcomes were collected. Tissue samples were interrogated using the Oncomine Comprehensive Assay v3 (ThermoFisher). Kaplan-Meier method was used to estimate the disease-free survival and overall survival (OS).

**Results:**

Overall, 11 patients with NSR-SCCUB were identified between 2002 and 2017 with adequate tissue samples. Median age was 71 years (45–86), predominantly male (63.6%). At time of diagnosis, 9 patients (81.8%) had muscle-invasive disease, 1 (9.1%) had non-muscle invasive, and 1 (9.1%) had advanced disease. Nine (81.8%) patients had radical cystectomy and pelvic lymph nodes dissection. Eight (72.7%) patients had pT3 or pT4 with N0, and 5 (45.5%) were grade 3. Median OS was 12.5 months (95% CI 7.7–17.2 months). Single nucleotide variants or insertion/deletions were identified in *TP53*, *TERT*, *PIK3CA*, *PTEN*, *CREBBP*, FBXW*7*, *and FGFR3*. Amplifications were found in *CCND1*, and *EGFR*.

**Conclusions:**

NSR-SCCUB has potentially actionable genomic alterations with anticancer agents and many of these aberrations are also seen in UC. The recruitment of NSR-SCCUB patients harboring such mutations should be considered in biomarker driven urinary bladder cancer studies.

## Background

Bladder cancer is the leading cause of the urinary tract malignancy and the fifth most common cancer in Canada with 12,200 cases diagnosed in 2020 [1]. Urothelial carcinoma (UC) represents the most common histologic subtype of bladder cancer (~90% of cases), with other rare subtypes including squamous cell carcinoma (SCC) (2–5%), adenocarcinoma (0.5–2%), and small cell carcinoma (<1%). Although mixed urothelial and non-urothelial bladder cancers can occur, the diagnosis of pure SCC requires the absence of any urothelial component [2–4]. Pure SCC of the urinary bladder is rare, but in regions where schistosomal infections are endemic such as Egypt and Japan, SCC is diagnosed at a higher frequency with some series reporting up to 60% [5–7].

Non-schistosomiasis related-squamous cell carcinoma of urinary bladder (NSR-SCCUB) is epidemiologically and biologically distinct from schistosomiasis related-SCC of the urinary bladder (SR-SCCUB) [4, 8]. The NSR-SCCUB type is more prevalent in western countries like Europe, USA, and Canada; has slightly higher male predominance and is linked to chronic bladder irritation (cyclophosphamide chemotherapy, or chronic bladder infections, indwelling catheters). Furthermore, NSR-SCCUB tumors tend to have advanced age, T stage at diagnosis (≥T2), affecting the trigone or lateral walls of the bladder. They are poorly differentiated and have a higher histological grade and necrosis percentage but lower incidence of both lymphovascular invasion and lymph node metastasis. Distant metastases occur in approximately 10–30% of cases of muscle-invasive SCC, but local and pelvic recurrences remain the major cause of relapse and death [4, 9–12].

Radical cystectomy and pelvic lymph node dissection with urinary diversion is the only curative treatment modality for localized muscle-invasive disease [9, 13]. Adjuvant radiation may improve disease-free survival (DFS) compared to radical cystectomy alone, but this is not standard practice [14]. To date, there is insufficient evidence to support neoadjuvant or adjuvant chemotherapy in patients with localized disease [14–16].

Currently, no molecular biomarkers exist in NSR-SCCUB. Notably, differences in clinical behaviour and outcomes between SR-SCCUB and NSR-SCCUB suggest that these are clinically distinct entities. In our study, we aimed to go beyond a clinico-pathological explanation and describe the genomic landscape of NSR-SCCUB.

## Material and methods

### Patients and data collection

Patients with NSR-SCCUB diagnosed between January 2002 and January 2017 at Princess Margaret Cancer Centre were identified retrospectively from central pathology records. Clinical characteristics such as age, gender, disease risk factors, pathology, radiology, cystoscopic findings (size and location of tumor), treatment modalities (radiotherapy, chemotherapy, surgery), and survival outcomes were collected. Histopathology slides (hematoxylin and eosin–stained slides, special stains, and immunohistochemistry) of all cases were reviewed and verified by specialized genitourinary pathologists. Histological type, grade, muscle invasion, necrosis, lymphovascular involvement and lymph node status were recorded. Samples were subsequently processed for Next Generation Sequencing (NGS) analysis. Our study has received an approval from the university health network institutional ethics committee (#17–6072) and conformed to the declaration of Helsinki.

### Nucleic acid extraction

Tumor DNA and RNA were extracted from 5μm-thick formalin-fixed paraffin-embedded (FFPE) tissue sections based on annotation of a corresponding H&E slide by a pathologist. Samples from either radical cystectomy specimens with muscle invasive bladder cancer or transuretheral resection of bladder tumors (TURBT) were used. Areas were chosen to ensure a 70% minimum tumor cell proportion within the selected region. Extraction was performed by using the Maxwell RSC RNA FFPE kit to isolate total nucleic acids on an automated Maxwell 16 Research extraction system (Promega, Madison, USA). The Maxwell RNase A and DNase I solutions were used to isolate RNA and DNA, respectively, during the two different procedures of nucleic acids isolation. Nucleic acid quantification was performed by the Qubit 2.0 Fluorometer (Thermo Fisher Scientific).

### DNA and RNA NGS and data analysis

NGS library preparation for the Oncomine Comprehensive Assay v3 (OCAv3, Thermo Fisher Scientific, Waltham, MA, USA; catalog number, A35805) using extracted DNA and RNA was performed using the Ion AmpliSeq Library Preparation on the Ion Chef System (Thermo Fisher Scientific). Sequencing was performed on the IonTorrent™S5 XL platform, following manufacturer protocols. OCAv3 is an amplicon-based, targeted assay that enables the detection of relevant single nucleotide variants and small insertions and deletions in gene hotspots and full coding regions, amplifications, and gene fusions from 161 unique genes. Genomic data were analyzed, and alterations were detected using the Ion Reporter software, version 5.6 (Thermo Fisher Scientific). No non-cancerous tissue was analyzed however genetic polymorphisms were removed by querying the gnomAD database v 2.1.1 [17, 18].

### Statistical analysis

Clinical and treatment characteristics were reported descriptively. For survival calculations, time from pathologic diagnosis to event of interest was used for DFS [recurrence] and overall survival (OS) [death from any cause]. Survival estimates for DFS, and OS were performed using the Kaplan-Meier method. Statistical analyses were performed using IBM SPSS Statistics v24 (IBM; Armonk, NY, USA).

## Results

### Patient characteristics

Between 2002 and 2017, 15 patients with NSR-SCCUB were identified but only 11 had adequate pathology samples. Nine radical cystectomy and two TURBT samples were used. Their median age was 71 years (range 46–86), seven (63.6%) patients were male and five (45.4%) were smokers. Six (54.5%) patients had irritant exposure (stone, infection, radiation, or catheter). One (9.1%) patient had a chronic in-dwelling catheter; three (27.3%) patients had prior pelvic irradiation, two (18.2%) had prostate cancer and one (9.1%) had ovarian cancer. Three (27.3%) patients had evidence of chronic urinary tract infection from either fungus, human papillomavirus, or bacteria. Urinary diverticulum was present in 2 (18.2%) patients. One (9.1%) patient had chronic urinary bladder stones. A full description of their characteristics is outlined in (Table 1).

**Table 1:** Patient demographics, treatment modalities, and outcomes (N = 11).

Variables	Patients number (%)
Age (years), median [IQR]	71 [46–86]
Gender	
Male	7 (63.6)
Female	4 (36.4)
Smoking	
No	6 (54.5)
Yes	5 (45.5)
Irritation/inflammation	
No	5 (45.5)
Yes	6 (54.5)
Urinary Tract Infection Hx	
No	8 (72.7)
Yes	3 (27.3)
Past history of malignancy	3 (27.3)
Prostate Cancer	2 (18.2)
Ovarian Cancer	1 (9.1)
Previous history of pelvic radiotherapy	3 (27.3)
Radical Cystectomy & PLND	
No	2 (18.2)
Yes	9 (81.8)
Adjuvant chemotherapy	
No	7 (63.6)
Yes	2 (18.2)
NA	2 (18.2)
Adjuvant radiotherapy	
No	8 (72.7)
Yes	1 (9.1)
NA	2 (18.2)
Local relapse	
No	7 (63.6)
Yes	2 (18.2)
NA	2 (18.2)
Systemic relapse	
No	6 (54.5)
Yes	3 (27.3)
NA	2 (18.2)
First Line Chemotherapy in M1	
No	8 (72.7)
Yes	3 (27.3)

Abbreviations: Hx, history; PLND, pelvic lymph node dissection; NA, not applicable; M1, metastatic stage.

The most common presenting symptoms were gross hematuria in 8 (72.7%) patients, followed by irritative bladder symptoms (e.g., dysuria, suprapubic pain, frequency, and urgency) in 6 (54.5%) patients. All patients underwent cystoscopy that identified 5 (45.5%) tumors on the lateral wall, 3 (27.3%) on the posterior wall, 3 (27.3%) at the trigone and 2 (18.2%) at the bladder base. All patients underwent transurethral resection of a bladder tumour that confirmed the diagnoses of NSR-SCCUB. Nine (81.8%) patients had muscle-invasive disease and 1 (9.1%) had non-muscle invasive tumor, while the remaining patient had metastatic disease to lungs, lymph nodes, peritoneum, and brain at presentation. Nine (81.8%) patients underwent radical cystectomy and lymph node dissection. The tumor size, grade, margins, lymph vascular invasion, presence of necrosis, TNM stage, site of relapses, site of metastasis and treatment modalities are outlined in (Tables 2 and S1).

**Table 2:** Tumor pathology characteristics.

Variables	Patients number (%)
**Stage**	
Superficial	1 (9.1)
MIBC	9 (81.8)
Advanced	1 (9.1)
**T-size, median [IQR]**	156 ml [19–584]
**T-stage**	
≤T2	2 (18.2)
T3	4 (36.3)
T4	5 (45.5)
**Node**	
Negative	8 (72.7)
Positive	2 (18.2)
Unknown	1 (9.1)
**Grade**	
2	6 (54.5)
3	5 (45.5)
**Margins**	
Negative	6 (54.5)
Positive	3 (27.3)
Unknown	2 (18.2)
**Lymph vascular Invasion**	
Negative	8 (72.7)
Positive	2 (18.2)
Unknown	1 (9.1)
**Necrosis**	
Absent	8 (72.7)
Present	2 (18.2)
Unknown	1 (9.1)

Abbreviations: MIBC, Muscle Invasive Bladder Cancer

### Survival and genomic analyses

**Fig 1:**
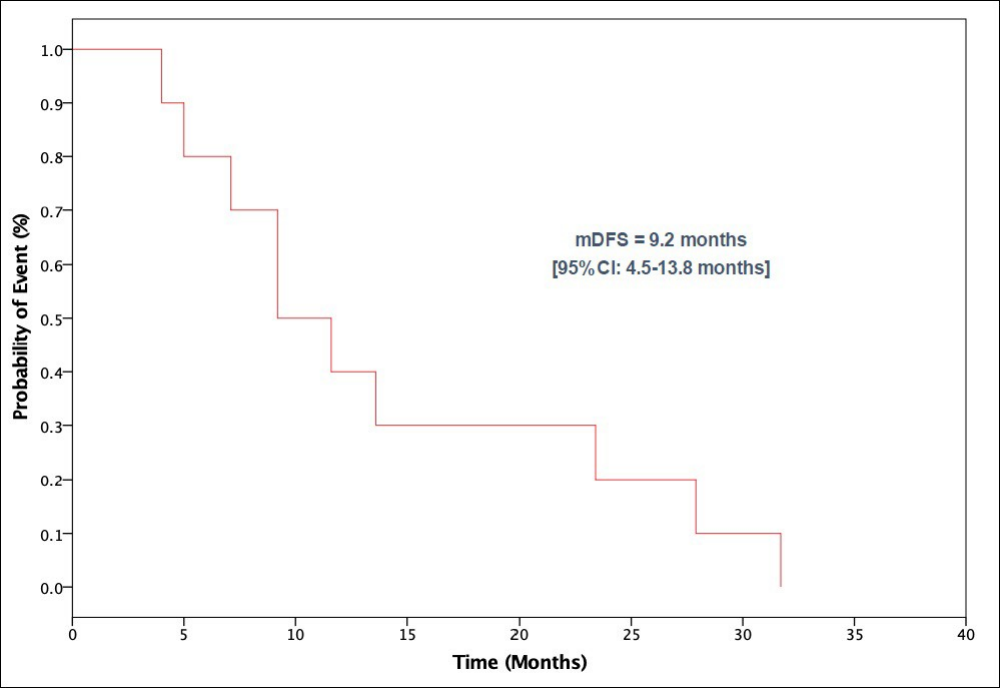
Kaplan Meier curve of disease-free survival for non-metastatic patients. Abbreviations: mDFS, median disease-free survival.

**Fig 2:**
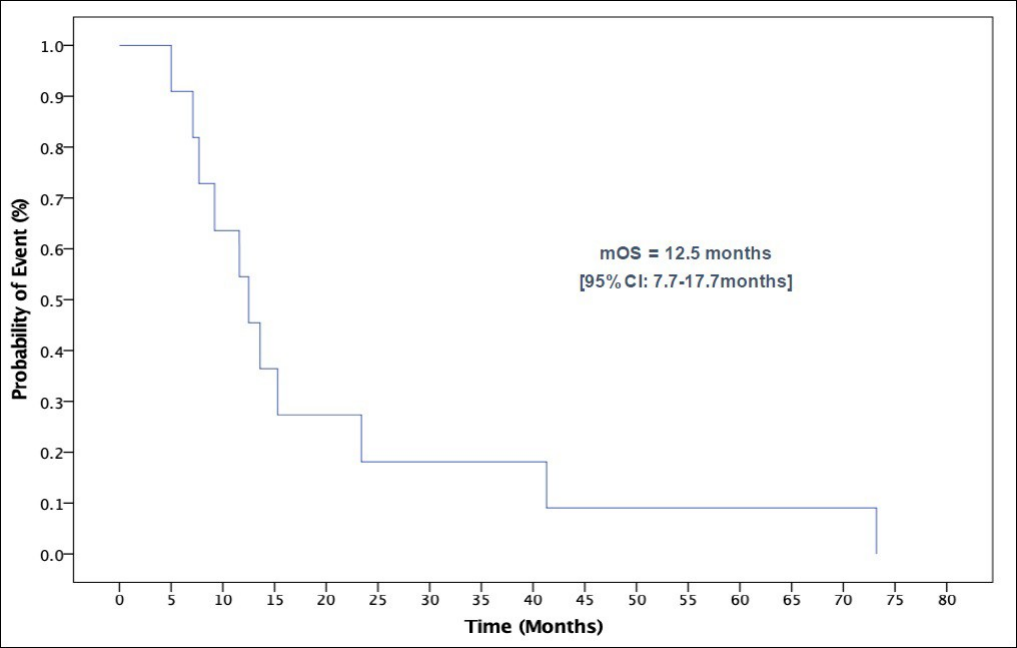
Kaplan Meier curve of overall survival for all patients. Abbreviations: mOS, median overall survival.

In the entire cohort, median DFS, excluding one patient who has metastatic disease at presentation, was 9.2 months (95%CI: 4.5–13.8 months) (Fig 1). Median OS was 12.5 months (95% CI 7.7–17.2 months) (Fig 2).

**Fig 3:**
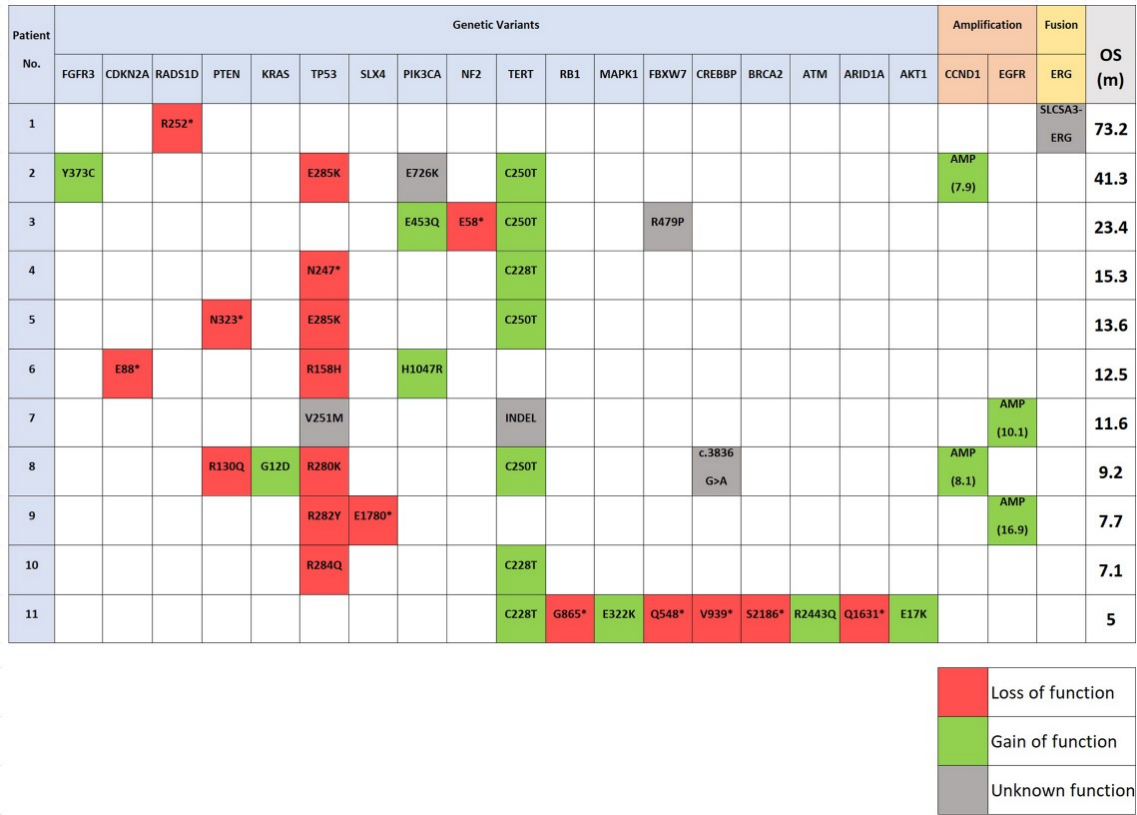
The molecular heatmap of the genomic alterations and survival durations arranged based on patient number. Abbreviations: No., number; AMP, amplification (number of copies); OS, overall survival; m, months; *, termination (stop) codon.

Common variants were identified in *TERT* (72.7%), *TP53* (72.7%), *PIK3CA* (27.3%), *CREBBP* (18.2%), *FBXW7* (18.2%), and *PTEN* (18.2%) genes. Amplifications were identified in *EGFR* (18.2%), and *CCND1* (18.2%). Additional variants were detected at a frequency of <10% in genes such as *FGFR3*, *CDKN2A*, *RADS1D*, *KRAS*, *SLX4*, *NF2*, *RB1*, *MAPK1*, *BRCA2*, *ATM*, *ARID1A*, and *AKT1*. RNA was analyzed for all 11 cases. Due to suboptimal RNA, 7 of the 11 failed our lab’s quality control (QC) metrics. Of the four cases that passed QC metrics, a *SLCSA3-ERG* fusion was detected in only one sample (9.1%) with unknown significant function (Fig 3 and Table 3).

**Table 3:** The most common gene type, frequency, location, category, and the effects of variants on the gene function in our cohort.

Gene type	Frequency (%)	Gene location	Category	Gene function	Variant’s frequency in our cohort with effects on the gene function					
					Gain of function	N	Loss of function	N	Unknown function	N
*TERT*	8 (72.7%)	5p15.33	Oncogene	Encodes Telomerase that maintain telomere length and genomic stability [19].	C250T	4	NA		c.-138_139cc>TT INDEL	1
					C228T	3				
*TP53*	8 (72.7%)	17p13.1	Tumor suppressor protein	Negative regulator of cell proliferation and a positive regulator of apoptosis in response to DNA damaging agents [20].	NA		E285K	2	V251M	1
							R280K	1		
							R284Q	1		
							R282Y	1		
							R158H	1		
							N247*	1		
*PIK3CA*	3 (27.3%)	3q26.32	Oncogene	Phosphorylates certain signaling molecules, that trigger many cell activities, including cell growth, proliferation, migration of cells, production of new proteins, transport of materials within cells, and cell survival [21].	E453Q	1	NA		E726K	1
					H1047R	1				
*FBXW7*	2 (18.2%)	4q31.3	Tumor suppressor gene	It is a member of the F-box protein family, which is part of the Skp1-Cdc53/Cullin-F-box-protein complex (SCF/β-TrCP). The SCF complex is an E3-ubiquitin ligase that ubiquitinates proteins and triggers proteasome degradation [22].	NA		Q458*	1	R479P	1
*CREBBP*	2 (18.2%)	16p13.3	Tumor suppressor gene	It involves in the transcriptional coactivation of many different transcription factors [23].	NA		V939*	1	c.3836 G>A T1279I	1
*PTEN*	2 (18.2%)	10q23.31	Tumor suppressor gene	It triggers signals stop growth and self-destruct (apoptosis) [24].	NA		N323*	1	NA	
							R130Q	1		
*EGFR*	2 (18.2%)	7p11.2	Oncogene	It promotes cell growth, division, and cell survival [25]	AMP 10.1 copies	1	NA		NA	
					AMP 16.9 copies	1				
*CCND1*	2 (18.2%)	11q13.3	Oncogene	Encodes a protein that regulate subunit of CDK4 or CDK6, which required for cell cycle G1/S transition. It interacts with tumor suppressor protein Rb [26]	AMP 7.9 copies	1	NA		NA	
					AMP 8.1 copies	1				

Abbreviations: NA, not applicable; AMP, amplification;

*, termination (stop) codon.

## Discussion

Our retrospective analysis identified well known risk factors for NSR-SCCUB such as smoking history (45.5%), urinary bladder irritant exposure (bladder stone, chronic urinary tract infection [fungus, human papillomavirus, or bacteria], prior pelvic radiotherapy, chronic indwelling catheter) (54.5%) and the presence of urinary diverticulum (18.2%). Our clinical findings corroborate Manely et al’s report from a retrospective multicenter study of 90 patients with NSR-SCCUB who found smoking (27.8%), recurrent urinary tract infection (20.0%), indwelling catheter use (13.3%), neuropathic bladder (10.0%), bladder stones (3.3%), bladder diverticulum (3.3%) were risk factors for NSR-SCCUB [27]. Our cohort is too small to make robust conclusions and our findings would need confirmation in a larger population of patients. The role of screening for those with these risk factors is yet to be defined and could be explored further.

The population included in our cohort is representative of the typical patient with NSR-SCCUB and our analysis confirmed their poor outcomes with median overall survival of one year. There is an urgent need for more therapeutic options for these patients by analyzing their tumor for molecular alterations.

Our genomic analysis supported previous reports of NGS in NSR-SCCUB patients. *TERT* gene variants (72.7%) occurred at a high frequency which is consistent with the findings of Cowan et al in a group of patients with NSR-SCCUB post radical cystectomy. High rates of *TERT* gene variants (80%) are common in bladder SCC but have also been detected in UC with squamous differentiation and small cell carcinoma of the bladder. This shared genetic alteration between variant histologies of bladder carcinoma support the idea of a common oncogenic pathway for these tumors [19].

Our identification of *TP53*, *PIK3CA*, *FBXW7*,*and BRCA2* gene variants results are consistent with Geynisman et al who analyzed 24 NSR-SCCUB specimens using NGS and found gene variants in *TP53* (72.7%), *PIK3CA* (21.4%), *HRAS* (18.2%), *BRCA1* (16.7%), *BRCA2* (16.7%), and *FBXW7* (9.1%) [28]. Anari et al reported NGS results of 15 patients with NSR-SCCUB with the highest variant rate were *TP53* (66.7%), *PIK3CA* (33.3%), *HRAS* (14.3%), *FBXW7* (6.7%) and *AKT1* (6.7%) [29].

In terms of gene amplifications, we reported that *EGFR*, and *CCND1* were both increased in 18.2% of our cohort. Guo et al analyzed 16 cases of NSR-SCCUB and showed all were positive for EGFR expression by immunohistochemistry [30]. Interestingly, Millis et al analyzed 11 cases of NSR-SCCUB using fluorescence *in situ* hybridization (FISH). They identified one patient (9.1%) with EGFR amplification: ≥ 4 copies in ≥ 40% of tumor cells [31]. Zaharieva et al analyzed 33 samples of NSR-SCCUB with FISH and found amplifications in *CCND1* in 5 (15.2%) samples [32].

In our study we identified several actionable genetic mutations, raising the possibility that such patients could be enrolled on clinical trials testing drugs that target these aberrations. Our series confirmed the poor prognosis that most of these patients have highlighting the urgent, unmet need to develop new therapeutic strategies for patients with NSR-SCCUB. In this study, we demonstrated that half the relapses were local while the other half were distant failures. This suggests that these patients could be considered for clinical trials for intensified therapy in the localized setting, perhaps with perioperative radiation and or chemotherapy.

The FGFR inhibitor, erdafinitib is approved by the US Food and Drug Administration (FDA) for the treatment of patients with metastatic urothelial carcinoma who have progressed on platinum-based chemotherapy and who harbor aberrations in *FGFR2* and *FGFR3* genes [33]. In attempt to identify *FGFR* genetic variants in patients with advanced UC who may have most benefit from erdafitinib treatment, Wang et al analyzed *FGFR* gene variant positive patients’ outcomes from the clinical trial assay used in the phase 2 study that approved erdafitinib. They identified 7 out of 11 patients with a *FGFR* gene variant (Y373C) with overall response rate on erdafitinib of 63.6% (95%CI: 35.4–84.8) [34]. We identified such abnormality in one of the patients enrolled in this study and thus FGFR inhibitors may represent a potential treatment option. Typically, patients with non-urothelial bladder cancers are not included in the majority of clinical trials for patients with metastatic bladder cancer but we would argue that such patients who harbor the molecular aberration of interest, should be considered for enrollment in these biomarkers driven studies.

Other genomic aberrations identified include *PIK3CA* and *PTEN*, which may be actionable with PI3K, AKT or MTOR inhibitors. *PIK3CA* gene variants were associated with improved recurrence-free survival and improved cancer-specific survival in patients with UC treated with radical cystectomy [35]. We identified two *PIK3CA* gene variants (H1047R and E453Q) in our cohort. Janku et al reported that certain PIK3CA mutation can predict response to PI3K/AKT/mTOR signaling pathway inhibitors in early phase clinical trials patients with diverse advanced solid tumors. Patients with a *PIK3CA* H1047R variant compared to patients with other *PIK3CA* gene variants or patients with wild-type *PIK3CA* treated on the same protocols had a higher partial response rate. On multivariate analysis *PIK3CA* gene variant (H1047R) was the only independent factor predictive of response (odds ratio 6.6, 95% CI 1.02–43.0, p = 0.047) [36].

Anti-EGFR targeted therapy was extensively studied in advanced urinary bladder cancer. Rose et al characterized the genetic and protein expression levels in pure primary SCC (n = 75) and mixed squamous cell carcinoma (n = 50) of the urinary bladder and performed functional pathway and drug-response analyses with cell line models and isolated primary SCC cells of the human urinary bladder. EGFR expression was identified in 95% of the study cohort without evidence for activating *EGFR* mutations. *EGFR* by FISH revealed an amplification of the *EGFR* gene in 8%. Both mixed and primary SCC cells were sensitive to EGFR tyrosine kinase inhibitors (erlotinib and gefitinib) [37]. These findings give further support to investigate the role of anti-EGFR TKIs in treatment of patients with NSR-SCCUB.

*TERT* gene variants were a frequent abnormality found in just under 3 quarters of our cohort. This finding is comparable to what has been demonstrated in conventional UC, although this variant is not yet considered druggable. A recent metanalysis reported by Wan et al analyzed the prognostic implications of *TERT* gene variants, specifically C288T and C250T in conventional UC. Eight studies contained 1382 patients with 62% *TERT* gene variants. The study showed a significant correlation between *TERT* gene variants and risk of recurrence. However, there was no significant association with OS [38]. *TP53* gene variants were reported in our study at similar frequency to *TERT* gene variants. *TP53* genetic variants were reported in SR-SCCUB and conventional UC and were associated with inferior survival outcomes in both histologies [39, 40].

Most SCC studies that assessed the molecular landscape have been limited to SR-SCCUB or were conducted on a patient population that was enriched for SR-SCCUB. Some studies reported high levels of expression involving TP53 (~40–70%), EGFR (~70–100%), and HER2 (~30–60%) proteins [7, 39, 41, 42].

Studies have identified TP53 and FGF2 overexpression as predictive of progression and inferior overall survival [39, 43]. In one study, HER2 expression correlated with poor prognosis in both UC and SCC; however, all SCC patients were SR-SCCUB [39]. Other studies that reported a high frequency of HER2 expression did not examine its influence on outcomes [7, 44]. Youssef et al. found a correlation between FGF2 overexpression and aggressive pathologic features (lymphovascular invasion and nodal involvement). Furthermore, FGF2 overexpression predicted for poor prognosis. In addition, the authors reported an association between cyclooxygenase-2 expression and higher grade and stage at diagnosis, and a correlation with the poor outcome in SR-SCCUB [8].

Our study had limitations. The number of samples included in the analysis were small given the rarity of the disease, and our conclusions are therefore only hypothesis generating. The mean age of the samples was 13 years with range (4–21); therefore, the RNA quality was degraded preventing sequencing analysis in some cases. Finally, the NGS performed only covered a targeted gene panel, and thus a deeper analysis via whole exome or whole genome sequencing may have revealed other genomic abnormalities that we did not identify.

## Conclusions

NSR-SCCUB has potentially actionable genomic variants with targeted therapies. Many of these variants have been previously identified in UC. The recruitment of NSR-SCCUB patients harboring such genomic variants should be considered in biomarker driven urinary bladder cancer studies.

## Supporting information

S1 Table TNM stage, site of relapses, site of metastasis and treatment modalities arranged by patient number. (DOCX)

## Abbreviations

NSR-SCCUB: non-schistosomiasis related-squamous cell carcinoma of urinary bladder
UC: Urothelial carcinoma
SCC: squamous cell carcinoma
SR-SCCUB: schistosomiasis related- squamous cell carcinoma of urinary bladder
DFS: disease-free survival
NGS: next generation sequencing
OS: overall survival
