# Supplementary material for: Genomic characterization of non-schistosomiasis-related squamous cell carcinoma of the urinary bladder: A retrospective exploratory study
Source: PLoS One. 2021 Dec 1;16(12):e0259272. doi: 10.1371/journal.pone.0259272 (PMC8635362; doi:10.1371/journal.pone.0259272)
Supplement: S1 Table — (DOCX) [file pone.0259272.s001.docx]

**S1 Table**. TNM stage, site of relapses, site of metastasis and treatment modalities arranged by patient number.

| **Pt  no.** | **Age**  **(y)** | **Gender** | **Risk**  **Factors** | **Neo-adjuvant**  **therapy** | **type of surgery** | **TNM** | **Adjuvant Therapy** | **Relapse Site** | **Salvage  Therapy** | **Site of metastasis** | **Palliative  Therapy** |
| --- | --- | --- | --- | --- | --- | --- | --- | --- | --- | --- | --- |
| 1 | 69 | M | Smoking history, Previous history of pelvic radiotherapy for prostate cancer | No | RC&  PLND | pT4N0M0 | No | Perineal skin | Surgery | Lungs | Carboplatin and  gemcitabine |
| 2 | 69 | M | Smoking history, indwelling catheter, and fungal cystitis | No | TURBT | cT1N0M0 | BCG | Extensive Local | Cisplatin/XRT | No | No |
| 3 | 65 | F | Previous history of pelvic radiotherapy for ovarian cancer | No | RC&  PLND | pT3bN0M0 | No | No | No | No | No |
| 4 | 74 | F | Recurrent bacterial cystitis | No | RC&  PLND | pT3BN2M0 | No | No | No | LN, Liver,  lungs | No (PS4) |
| 5 | 86 | M | Previous history of pelvic radiotherapy for prostate cancer and bladder diverticulosis | No | RC&  PLND | pT3BN0M0 | No | No | No | No | No |
| 6 | 46 | F | No risk factors | No | RC&  PLND | pT4BN0M0 | Cisplatin  /XRT | NA | No | Iliac conduit, right inguinal LN, abdominal wall | Cisplatin and  Gemcitabine |
| 7 | 73 | M | Smoking history | No | RC&  PLND | pT4AN0M0 | No | No | No | No | No |
| 8 | 61 | M | Smoking history | No | RC&  PLND | pT3BN0M0 | Cisplatin  Gemcitabine | No | No | No | No |
| 9 | 50 | M | Smoking history  HPV cystitis  Urinary bladder stone | No | NA | cT2N3M1 | No | NA | NA | LN, peritoneum, brain and lungs | Cisplatin and  Gemcitabine, Palliative surgery and WBRT |
| 10 | 86 | F | No risk factors | No | RC&  PLND | pT4N0M0 | No | No | No | No | No |
| 11 | 77 | M | Bladder diverticulum | No | RC&  PLND | pT4aN0M0 | No | No | No | No | No |

Abbreviations: Pt no., patient number; M, male; F, female; RC&PLND, radical cystectomy and pelvic lymph node dissection; TURBT, transurethral resection of a bladder tumour; NA, not applicable; BCG, Bacillus Calmette–Guérin; XRT, radiotherapy; PS, performance status; LN, lymph node; WBRT, whole brain radiotherapy.
